# User-Guided Enhancements to a Technology-Facilitated Resilience Program to Address Opioid Risks Following Traumatic Injury in Youth: Qualitative Interview Study

**DOI:** 10.2196/45128

**Published:** 2023-11-30

**Authors:** Zachary W Adams, Brigid R Marriott, Swathi Karra, Elizabeth Linhart-Musikant, Jodi L Raymond, Lydia J Fischer, Kristina A Bixler, Teresa M Bell, Eric A Bryan, Leslie A Hulvershorn

**Affiliations:** 1 Department of Psychiatry, Indiana University School of Medicine Indianapolis, IN United States; 2 Riley Hospital for Children at Indiana University Health Indianapolis, IN United States; 3 Department of Surgery, University of Utah School of Medicine Salt Lake City, UT United States; 4 Department of Population Health Sciences, University of Utah School of Medicine Salt Lake City, UT United States

**Keywords:** access-to-care, addiction, adolescent, behavior, health disorder, opioid use disorder, opioid, personalized care, telehealth, telemedicine, trauma, user, youth

## Abstract

**Background:**

Youth with traumatic injury experience elevated risk for behavioral health disorders, yet posthospital monitoring of patients’ behavioral health is rare. The Telehealth Resilience and Recovery Program (TRRP), a technology-facilitated and stepped access-to-care program initiated in hospitals and designed to be integrated seamlessly into trauma center operations, is a program that can potentially address this treatment gap. However, the TRRP was originally developed to address this gap for mental health recovery but not substance use. Given the high rates of substance and opioid use disorders among youth with traumatic injury, there is a need to monitor substance use and related symptoms alongside other mental health concerns.

**Objective:**

This study aimed to use an iterative, user-guided approach to inform substance use adaptations to TRRP content and procedures.

**Methods:**

We conducted individual semistructured interviews with adolescents (aged 12-17 years) and young adults (aged 18-25 years) who were recently discharged from trauma centers (n=20) and health care providers from two level 1 trauma centers (n=15). Interviews inquired about reactions to and recommendations for expanding TRRP content, features, and functionality; factors related to TRRP implementation and acceptability; and current strategies for monitoring patients’ postinjury physical and emotional recovery and opioid and substance use. Interview responses were transcribed and analyzed using thematic analysis to guide new TRRP substance use content and procedures.

**Results:**

Themes identified in interviews included gaps in care, task automation, user personalization, privacy concerns, and in-person preferences. Based on these results, a multimedia, web-based mobile education app was developed that included 8 discrete interactive education modules and 6 videos on opioid use disorder, and TRRP procedures were adapted to target opioid and other substance use disorder risk. Substance use adaptations included the development of a set of SMS text messaging–delivered questions that monitor both mental health symptoms and substance use and related symptoms (eg, pain and sleep) and the identification of validated mental health and substance use screening tools to monitor patients’ behavioral health in the months after discharge.

**Conclusions:**

Patients and health care providers found the TRRP and its expansion to address substance use acceptable. This iterative, user-guided approach yielded novel content and procedures that will be evaluated in a future trial.

## Introduction

The present opioid epidemic is a serious public health crisis in the United States. In 2019, approximately 2.3% of adolescents aged 12-17 years and 5.3% of young adults aged 18-25 years misused opioids [1]. In recent reports, roughly half of all teens believed prescription drugs were much safer than illegal street drugs [2]. Importantly, people who become dependent on opioids are 40 times more likely to develop a heroin addiction, with 80% of heroin users reporting earlier misuse of prescription medications [3]. Nonmedical use of opioids and other prescription drugs tends to peak in late adolescence and early adulthood, calling for the implementation of preventative measures throughout adolescence [4-6].

Trauma is the leading cause of injury and death for children and adults under the age of 45 years in the United States [7]. Many patients with traumatic injury, including adolescents, receive prescription opioids at hospital discharge [8], heightening the risk for opioid use disorder (OUD). A nationally representative survey of high school seniors who reported nonmedical use of prescription opioids found 80% were first prescribed opioids by a physician [9]. One study found 52% of adolescents with traumatic injuries still consumed prescription opioids 2 months after discharge and 12.5% at 12 months after discharge [10]. Notably, youth with preexisting substance use or mental illness have been shown to be more vulnerable to poor behavioral health outcomes after injury, including an increased rate of opioid misuse and the likelihood of developing an opioid addiction [10,11].

Although substance use and mental health issues after a traumatic injury tend to affect quality of life, physical recovery, and the ability to return to previous activities, only 7% of trauma centers address these comorbid issues [12]. Hospitals rarely monitor patients’ behavioral health after discharge, missing opportunities to initiate treatment when it may be most effective [13]. For instance, among adults admitted to a local level 1 trauma center, less than 10% who screened positive for posttraumatic stress disorder (PTSD) and 22% for depression after injury received treatment for those disorders [14]. Taken together, these findings indicate a need for increased intervention efforts for young people who experience traumatic injury and use prescription opioids, particularly during the initial weeks after discharge.

The Telehealth Resilience and Recovery Program (TRRP) is a technology-facilitated, stepped-care program meant to address assessment and treatment gaps around PTSD and depression in youth and adults with traumatic injury [15]. TRRP activities include in-hospital education (step 1), enrollment in a “watchful waiting” daily SMS text message service to monitor emotional recovery (step 2), 30- and 60-day telephone mental health screens (step 3), and the provision of mental health treatment or referral (step 4) [15]. Leveraging technology in this way increases capacity for patient monitoring with minimal impact on provider workload, a key benefit given behavioral health workforce shortages [16]. A recent study of the TRRP examining data over 3 years found moderate to high patient engagement in each of the 4 intervention model steps among adults with traumatic injury [17]. The expansion and implementation of the TRRP in pediatric populations have also been shown to be feasible and increase access to services among those who need it [18,19].

Given the need for scalable interventions for patients who experience traumatic injury and are using prescription opioids, the TRRP may provide an intervention model that could facilitate education, monitor symptoms, and provide access to services for a wider range of behavioral health conditions, including substance use, for these patients. It is unknown whether the TRRP would be an acceptable or effective strategy for addressing opioid and other substance use in patients with traumatic injury. Thus, as a key step in this line of research, this study applied user-guided design principles to adapt TRRP content and procedures to target opioid and other substance use in addition to PTSD and depression. Qualitative interviews conducted with patients and health care providers from two level 1 trauma centers (ie, intended end users) informed the expansion of the TRRP. We present the qualitative findings from these interviews and describe the novel content and procedures that were ultimately developed.

## Methods

### Participants and Recruitment

Adolescent (aged 12-17 years) and young adult (aged 18-25 years) patients and trauma center health care providers were recruited for the study. Participants were recruited from 2 local, urban, level 1 trauma centers (1 exclusively pediatric and 1 that primarily treats adults and adolescents) affiliated with a large academic health center. Both sites treat a high volume of patients annually. Eligibility requirements for patients included being admitted and discharged from a local level 1 trauma center within the past 12 months. Health care providers were eligible if they provided care to patients who received care at a level 1 trauma center. All participants were required to have access to a smartphone, tablet, or computer to complete demonstrations and to be fluent in English since study materials were only available in English. Eligible patients for the study were identified from a list generated from the trauma registries at the two local level 1 trauma centers. The research team contacted eligible patients from the registries (and a parent or guardian if a minor) through telephone, SMS text message, recruitment postcard, or email to screen for eligibility and provide study information. To promote relevance and recall, priority was placed on recruiting patients who had been treated by each trauma center within the past 1-6 months when possible. Eligible providers were recruited by word of mouth and hospital daily staff newsletters. All participants completed verbal and written informed assent or consent procedures to participate in the study.

Researchers conducted interviews with a total of 35 participants. The sample included 9 adolescents (7 boys and 2 girls), 11 young adults (4 men and 7 women), and 15 trauma center health care providers. One patient interview was discontinued halfway through at the participant’s request due to a scheduling conflict. The most common causes of injury experienced by patients involved motor vehicle crashes (7/20, 35%) and firearm-related injuries (5/20, 25%). Health care professionals who participated in the study represented varied professional roles on the clinical team (Table 1).

**Table 1:** Characteristics of the patients of trauma centers and health care providers who participated in interviews.

Characteristics of patients and health care providers				Values
**Patients (n=20)**				
	Age (years), mean (SD; range)			17.6 (3.8; 12-15)
	**Sex**			
		Male, n (%)	11 (55)	
		Female, n (%)	9 (45)	
	**Type of Injuries, n (%)**			
		Motor vehicle crash	7 (35)	
		Firearm-related injuries	5 (25)	
		Pedestrian injuries	3 (15)	
		Falls	3 (15)	
		Sports-related	1 (5)	
		Knife wound	1 (5)	
**Provider profession (n=15), n (%)**				
	Registered nurse			6 (40)
	Nurse practitioner			3 (20)
	Licensed clinical social worker			2 (13)
	Physician			1 (7)
	Physical therapist			1 (7)
	Chaplain			1 (7)
	Program manager			1 (7)

### Procedures

The research team conducted semistructured interviews with patients and health care providers. Participants were given the option to be interviewed in-person, by videoconference, or by phone. All participants received US $50 e-gift cards for their participation. Interview questions were drafted based on user-guided design principles with the goal of eliciting rich, candid responses to guide further development of TRRP content [20]. In each interview, participants were provided a brief description of the current TRRP structure and content and then asked to provide their reactions and recommendations for new content, features, and functionality. Patients were also asked about their postinjury physical and emotional recovery, their experience with pain medications, and their education regarding the medication. Providers were asked about their current strategies for monitoring patients’ physical and emotional recovery following discharge from the trauma service and any concerns they had relating to opioid or other substance use in adolescent and young adult patients with traumatic injury. Additional questions addressed factors related to TRRP implementation and acceptability (eg, patient preference for how to be contacted and facilitators and barriers to participation).

All interviews were audio recorded and transcribed. Thematic analysis was used to identify categories or themes that captured the main ideas that emerged in the interview data [21]. Following Saldaña’s [21] guiding principles for qualitative data analysis, at least 2 researchers read through each transcription multiple times to identify and categorize observed codes in the interview responses. These categories were then reviewed to create emergent, higher-level themes. Through collaboration and consensus, the research team determined what statements belonged in each code, category, and theme. The research team continued searching for patterns until all the data were categorized and no new themes emerged.

### Ethical Considerations

This study was performed in line with the principles of the Declaration of Helsinki. The approval was granted by the Indiana University Institutional Review Board (protocol 2005652404). All participants completed verbal and written informed assent (for minors) or consent (for adults) procedures to participate in the study. All participants received US $50 e-gift cards for their participation. Interview transcripts were deidentified, password-protected, and stored on secure servers only accessed by trained research team members.

### Development of New Content and Procedures for the TRRP

To expand TRRP content and procedures to address opioid and substance use, specific web-based education content on OUD-related topics was developed as part of recovery education (ie, consistent with TRRP step 1). This content was developed based on the interview data and created through the collaboration of a multidisciplinary team. The team included adolescent behavioral health care specialists, a nonprofit organization dedicated to OUD and overdose prevention, a video production team, and an academic software development team. The health care and OUD experts created medically accurate, developmentally appropriate text on OUD-related topics and curated a list of pertinent local and national resources. The filmmaking team produced a series of 6 short videos addressing key elements of opioid education to complement the text content. The software development team built a secure web-based application to house the new educational content.

In addition to the educational content, a set of SMS text messaging–delivered questions was developed to help keep participants engaged with the program and to monitor patients’ behavioral health recovery (TRRP step 2). For each question, clinicians on the team drafted a brief set of tips or recommendations to promote healthy coping that were programmed to be sent to patients automatically after they responded to each daily question. Finally, validated mental health and substance use screening tools were identified to be administered to participants and monitor patients’ behavioral health in the months after discharge (TRRP step 3). These screening instruments were programmed to be completed on the internet or by phone based on patient preference.

## Results

### Qualitative Results

Most patients and health care providers noted that using a mobile app to provide education, address OUD risk, and monitor behavioral health recovery after trauma would be beneficial. For example, 1 patient stated, “...it’d be a good thing to have to be in contact with people who can access more help for me if I have any other issues or need assistance in any other ways. It would’ve been [a] nice tool to have.” In addition, several other themes emerged in the qualitative interviews, including gaps in care, personalized user preferences, in-person preferences, confidentiality concerns, and task automation. Responses were also classified based on relevance to each TRRP step.

### Gaps in Care

Both patients and health care providers identified current gaps in care with regard to education on the safety and risks of pain medication, education on emotional and behavioral recovery after traumatic injuries, and patient follow-up after discharge. Several patients reported being prescribed pain medications to manage their injuries but noted inconsistencies in the delivery of the education on their pain medication. For instance, some patients did not recall whether they received medication education, at times attributing poor recall to their condition while in the hospital (ie, incapacitated, cognitive impairment). Other participants reported that either they or their caregivers received medication education or counseling. Health care providers frequently noted a general lack of patient education on emotional and behavioral recovery in the hospital and follow-up after discharge, with most monitoring occurring at follow-up appointments or dependent on the patient or their caregivers to reach out. When asked about approaches to monitoring mood and anxiety, 1 provider stated, “There is none unless [a] child is experiencing it in the hospital, and we’ve set up follow-up appointments with child psychiatry and their PCP.”

### Education on Recovery (Step 1)

Regarding the first step of the TRRP, which is to provide education to patients and their families on recovering from traumatic injuries and the risks and safety related to pain medications, many patients felt it best to conduct initial education in-person while patients are still in the hospital instead of after discharge. One patient shared the following:

I feel like it’d be better for patients to have explanation by a live person in the hospital, so they know what to do and expect before they leave. That way they don’t have any worries about what to do at home.

Providers also indicated an app being useful for sharing education information with patients, especially after discharge, noting, “...You get a lot of papers when you go home from the hospital and it’s easy to lose those. Most have a smartphone and it’s pretty easy to get information from there if there’s an app.”

### Monitoring Recovery (Step 2)

Over half of patients reacted positively to the plan to send brief, daily SMS text messages to monitor emotional recovery for the first month following discharge from the hospital. One patient explained, “I would say that’d probably be good. I’d feel good that there was someone looking out for me. That’d help them feel better physically and emotionally.” Several providers also noted the acceptability of monitoring patients’ recovery after discharge, with 1 provider stating:

I think it would be really neat if you could have a text message that somehow reaches out to patients to see how they’re doing, or some sort of CBT type snippet or motivational thing to send out to those people, because I feel like they feel isolated and like the only ones who are going through

a traumatic injury

While patients had mostly positive reactions and indicated they would have been willing to sign up for the service, fewer patients stated they would respond to SMS text messages. Patients tended to favor responding to a small number of SMS text messages (ie, 1-6 questions) and spending a little amount of time (eg, ≤5 minutes) daily. Similarly, while some providers felt daily SMS text messages would be beneficial if patients had memory issues or brain injuries, others thought daily contact was unnecessary or excessive, potentially leading patients to drop out. For example, a provider reported:

That’s a tough one. I feel like we all get so many random text messages from all types of places…But I think there’s value in it. But I get worried that people are going to start ignoring them after the first couple days.

### Mental Health Screens (Step 3)

Researchers also queried participants on how to conduct the follow-up evaluations with patients. Participants favored conducting follow-up evaluations in-person due to being able to read the patient’s body language and convey care to patients. One patient noted, “I’d say in-person just because you can read somebody’s body language, where on the web you can’t really read them...” Other participants recommended using the app or providing a choice between methods (eg, phone, app, web-based, and in-person).

### Mental Health Treatment or Referrals (Step 4)

Additional themes of task automation and barriers to treatment referrals arose when asking patients and providers about connecting patients to treatment services if indicated. Transportation (eg, gas costs and automobile availability), treatment costs, and mobility limitations caused by certain injuries were commonly noted as barriers when referring patients to treatment services. A provider shared the following example:

Trying to find transportation is a big barrier. I see trauma folks, so their cars are often in accidents so they can’t drive them. Also, the financial issue of gas and parking and trying to figure out navigating downtown. All these obstacles before they get to the appointment. One recommendation cited by providers to increase the feasibility of the referral process was the automation of referrals to help staff who are overextended and unable to complete the task, particularly for patients who have endorsed low levels of symptoms.

### Confidentiality Concerns

Confidentiality concerns were also raised. Patients expressed concerns about replying to SMS text messages regarding medication or substance use, mood, pain, or sleep. Several patients articulated confidentiality concerns related to completing follow-up evaluations and treatment service referrals. For instance, 1 patient noted, “It’s all confidential, right? I would make sure that’s definitely said. Some of it is hard to talk about enough, let alone talking to someone you don’t know about it.”

### Personalized User Preference

Tailoring the program to individual preferences was another emergent theme. Patients suggested allowing a patient to personalize the time of day or number of SMS text messages they receive to increase its acceptability and feasibility. For instance, 1 patient stated:

Maybe once a day, every day would be a little excessive for the whole month. I’d say every day for a week or 2 after [discharge], and then maybe once or twice a week, based on how severe their injury is. Or give them the option of how often they’d want to have that interaction option.

Further, nearly all of the patients interviewed thought it would be helpful to receive personalized feedback or tips based on their responses to the SMS text messages.

### Updated TRRP Content and Procedures

**Figure 1:**
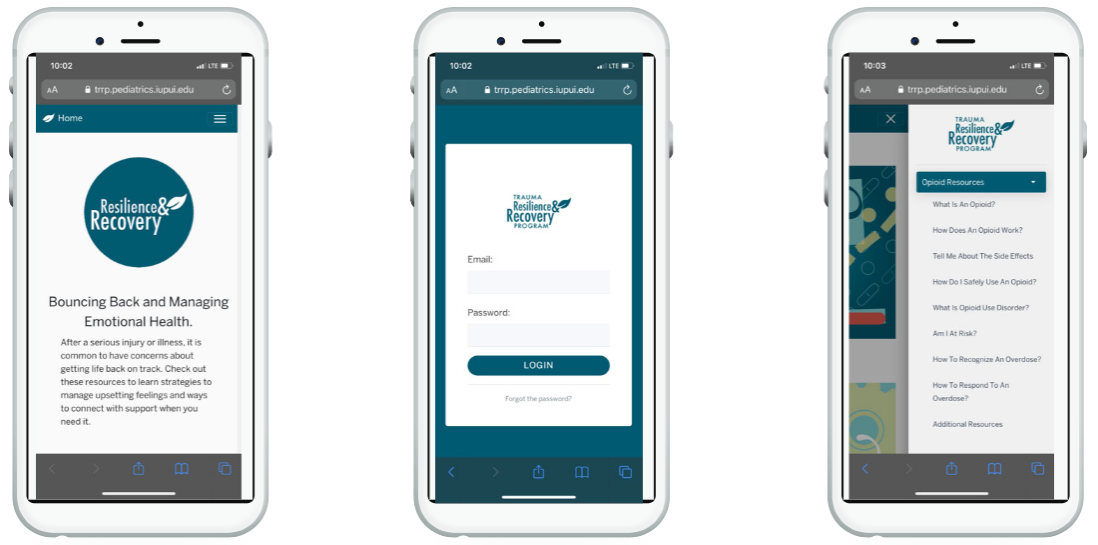
Screenshots of the multimedia, web-based, mobile education app.

**Figure 2:**
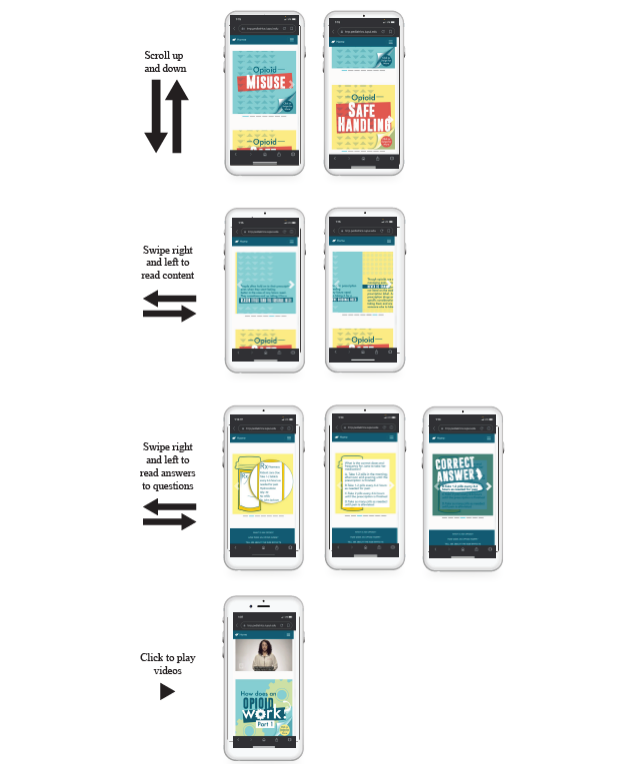
Example screenshots to demonstrate the functionality of the multimedia, web-based, mobile education app.

TRRP content and procedures were adapted to target opioid and substance use, in addition to mental health concerns. As noted in the *Methods* section, these adaptations were developed based on the themes that emerged during the qualitative interviews and through the collaboration of a multidisciplinary team. Textbox 1 outlines the specific topics for new content and modules created in this project. Each topic was translated into visually appealing and interactive (eg, multiple-choice questions and videos) learning modules that reside inside an easily navigable, password-protected, web-based application accessible on a computer or handheld mobile device (Figure 1). Examples of the interactivity can be found in Figure 2.

A technology-based screening protocol was developed that refined existing TRRP procedures to be tailored to the prevention of opioid and substance use in young people with trauma exposure. Once enrolled, in addition to the expanded education on recovery (TRRP step 1), participants complete brief but daily SMS text messaging–based check-ins (TRRP step 2) as well as a series of follow-up evaluations (TRRP step 3). For the SMS text messaging–based screenings, participants complete 1 SMS text messaging–based check-in daily for 60 days following discharge, with 12 total questions texted and repeated 5 times over the 60 days asking about their mood, anxiety, pain, sleep, and substance use. At 1-, 2-, 3-, and 6-month follow-up evaluations, patients fill out a series of validated mental health and substance use screenings along with a health care use survey (see Table 2 for selected screening tools). These assessments are brief, easily accessible, automated, and confidential and can be administered on the internet through a web-based survey or by phone. If participants screen positive, the clinical research team would be alerted to arrange a more thorough evaluation and referral for additional care when indicated.

**Newly developed opioid use disorder (OUD) education module topics.**

**Modules and their descriptions**

- Module 1: defines opioids, describes common uses of opioids, and explains the difference between synthetic opioids and opioids derived from the poppy plant.
- Module 2: explains how opioids work (written at a middle school level), describes how pain is experienced, and illustrates how opioid receptors impact the experience of pain
- Module 3: discusses short- and long-term side effects, including a brief introduction to OUD.
- Module 4: explains how to safely use an opioid and divides 3 topics into opioid misuse, safe-handling, and prescription label reading.
- Module 5: discusses OUD.
- Module 6: outlines risk and protective factors for OUD.
- Module 7: discusses how to recognize OUD in a developmentally appropriate way.
- Module 8: addresses how to respond when individuals overdose on opioids.

**Table 2:** List of identified mental health and substance use screening tools for follow-up evaluations.

Assessments	Description
Brief Pain Inventory, Short Form	9-item questionnaire that evaluates the severity and impact of the patient’s pain on a daily basis [22]
CAGE-AID^a^	4-item questionnaire that screens for lifetime use of alcohol and drug problems using a binary format [23]
Phen-X Toolkit Pediatric Quality of Life Enjoyment and Satisfaction Questionnaire	15-item questionnaire for children and adolescents (aged 6-17 years) that captures an individual’s general health, feelings, and well-being over the past week [24]
Patient Health Questionnaire-9	10 items that evaluate the level of depression an individual is experiencing [25]
Generalized Anxiety Disorder, 7-item	7-item questionnaire that screens for generalized anxiety disorder using a Likert scale [26]
Community Norms Prescription Drug Use Questionnaire	22 items that evaluate attitudes and awareness related to prescription drugs in the community
DSM-5^b^ Level 2—Substance Use—Child Age 11-17	15-item questionnaire assessing alcohol, nicotine, prescription medicine, and illicit drug use in children and adolescents [27]
Child PTSD^c^ Symptom Scale for DSM-5	27 items used to evaluate children and adolescents (aged 8-18 years) for PTSD and level of severity within the past month [28]

^a^CAGE-AID: Cut Down, Annoyed, Guilty, and Eye-Opener questionnaire.

^b^DSM-5: Diagnostic and Statistical Manual of Mental Disorders, 5th edition.

^c^PTSD: posttraumatic stress disorder.

## Discussion

### Overview

This study used qualitative methods to guide the expansion of the TRRP, an existing technology-facilitated, stepped-care program, to address opioid and other substance use in addition to PTSD and depression. Several common themes emerged in the interviews conducted with patients and health care providers from two level 1 trauma centers, including gaps in care, task automation, personalized user preference, confidentiality concerns, and in-person preferences. Regarding gaps in care, inconsistencies regarding patient education around prescription opioids as well as a lack of behavioral health monitoring and follow-up after discharge were noted. The TRRP is well suited to address these gaps in care and aims to educate patients about opioids by providing developmentally tailored videos on opioid-related topics. Moreover, the TRRP may provide a safety net for patients who might otherwise fall through the cracks after discharge by connecting them to a team that regularly assesses their behavioral health and can organize follow-up efforts. Future research should examine the effectiveness of the expanded TRRP developed in this study in increasing postdischarge behavioral health monitoring and follow-up and if it ultimately improves patient behavioral health outcomes and access to care, particularly with regard to opioid and other substance use. This is especially important given that many youth’s first exposure to opioids will come from a physician or other prescribing provider within the health care system [9,10], which for some patients may set the stage for the unintended development or exacerbation of a substance use disorder.

Participants also provided recommendations to improve the acceptability and feasibility of the TRRP. To increase its feasibility, providers frequently reported the need for task automation to reduce the burden on overwhelmed and overextended providers. Indeed, TRRP procedures are primarily technology-facilitated and entail task automation (eg, technology-based screenings), minimizing the burden on providers. Patients also suggested tailoring the TRRP toward individual preferences and addressing confidential concerns to improve acceptability. For example, patients indicated that participants should have the ability to opt out of daily SMS text messages and receive personalized tips or feedback based on how they respond to messages. Additionally, researchers should provide information and field questions about confidentiality during the informed consent process to ease patients’ concerns about responding to sensitive screening questions and using video technology to complete follow-up evaluations.

Participants also endorsed a preference for in-person recovery education and follow-up evaluations. However, it was not surprising that in-person interactions were favored by participants, who at the same time, were able to identify barriers to such care (eg, transportation costs and distance). It is exactly these barriers that make technology-based screening tools an ideal solution to provide access to quality mental health care for those who need it. While access to digital devices and internet connections present potential barriers to technology-based tools, a large majority of adolescents and young adults have reliable access to digital devices [29,30]. Furthermore, these interviews were conducted before the COVID-19 pandemic, which resulted in the burgeoning use of telehealth and technology-based tools in health care. In fact, the TRRP model transitioned to a fully remote model during COVID-19 [31]. Consequently, patient preferences toward in-person care may have shifted more recently [32].

### Limitations

One limitation of this study included only conducting the semistructured interviews in English, restricting the use of non-English participants. Future research should learn the preferences or procedures preferred by non-English speaking participants and include the translation of study materials into other languages. A second limitation is the possibility that participants may have provided socially acceptable or desirable responses. Third, it is possible that interviewing patients and providers in other locations and trauma centers may provide a different set of responses. Future studies should conduct interviews with a larger sample size of patients and providers from various locations to aid in the generalizability of the study results.

### Conclusion

This study expanded the content and procedures of the TRRP, an existing technology-facilitated, stepped-care program, to also address opioid and substance use in young people with trauma exposure. Findings from qualitative interviews illuminated common themes and recommendations to inform the adaptation of TRRP. This iterative and user-guided approach yielded a multimedia, web-based, mobile education app to be integrated within TRRP content. A pilot study is underway to evaluate the usability, acceptability, and initial efficacy of the newly developed TRRP content and procedures. Future work will address broad dissemination to increase accessibility of high-quality behavioral health services for young people affected by trauma.

## Abbreviations

OUD: opioid use disorder
PTSD: posttraumatic stress disorder
TRRP: Telehealth Resilience and Recovery Program

## Data Availability

The data sets generated during and analyzed during this study are available from the corresponding author on reasonable request and with appropriate approvals.
